# A HapMap leads to a *Capsicum annuum* SNP infinium array: a new tool for pepper breeding

**DOI:** 10.1038/hortres.2016.36

**Published:** 2016-07-27

**Authors:** Amanda M Hulse-Kemp, Hamid Ashrafi, Joerg Plieske, Jana Lemm, Kevin Stoffel, Theresa Hill, Hartmut Luerssen, Charit L Pethiyagoda, Cindy T Lawley, Martin W Ganal, Allen Van Deynze

**Affiliations:** 1 Department of Plant Sciences, University of California-Davis, Davis, California 95616, USA; 2 Illumina Incorporated, 5200 Illumina Way, San Diego, CA 92122, USA; 3 TraitGenetics GmbH, Am Schwabeplan 1b, Stadt Seeland OT, Gatersleben, Germany

## Abstract

The *Capsicum* genus (Pepper) is a part of the Solanacae family. It has been important in many cultures worldwide for its key nutritional components and uses as spices, medicines, ornamentals and vegetables. Worldwide population growth is associated with demand for more nutritionally valuable vegetables while contending with decreasing resources and available land. These conditions require increased efficiency in pepper breeding to deal with these imminent challenges. Through resequencing of inbred lines we have completed a valuable haplotype map (HapMap) for the pepper genome based on single-nucleotide polymorphisms (SNP). The identified SNPs were annotated and classified based on their gene annotation in the pepper draft genome sequence and phenotype of the sequenced inbred lines. A selection of one marker per gene model was utilized to create the PepperSNP16K array, which simultaneously genotyped 16 405 SNPs, of which 90.7% were found to be informative. A set of 84 inbred and hybrid lines and a mapping population of 90 interspecific F_2_ individuals were utilized to validate the array. Diversity analysis of the inbred lines shows a distinct separation of bell versus chile/hot pepper types and separates them into five distinct germplasm groups. The interspecific population created between Tabasco (*C. frutescens* chile type) and P4 (*C. annuum* blocky type) produced a linkage map with 5546 markers separated into 1361 bins on twelve 12 linkage groups representing 1392.3 cM. This publically available genotyping platform can be used to rapidly assess a large number of markers in a reproducible high-throughput manner for pepper. As a standardized tool for genetic analyses, the PepperSNP16K can be used worldwide to share findings and analyze QTLs for important traits leading to continued improvement of pepper for consumers. Data and information on the array are available through the Solanaceae Genomics Network.

## Introduction

The *Capsicum* genus in the Solanaceae family, commonly known as pepper or paprika, has been very important in many cultures worldwide for spices, medicines, ornamentals and vegetables and are key components of many food dishes. The fruits provide a high nutritional value and are a rich source of Vitamins A, B and C, iron, potassium, magnesium, beta carotene, folic acid and fiber. Cultivated peppers are typically divided into two categories, bell and chile. Bell peppers (or blocky type) refer to *C. annuum* varieties with blocky shaped fruits that are sweet (non-pungent) and can come in many colors from green (not fully ripe) to oranges, reds and purples. Whereas chile peppers (or hot types) refer to a large number of varieties that tend to have an elongated shape and can vary greatly in spice (pungency) from mild to extremely spicy, including ancho, anaheim, cayenne, fresno, habanero, jalapeno, poblano and serrano, just to name a few. The chile category contains types of all five domesticated species of *Capsicum*: *C. annuum*, *C. frutescens, C. chinense, C. pubescens, and C. baccatum.*

Production of these two pepper classifications, primarily composed of *C. annuum* varieties, totaled 44 800 acres of bell peppers and 19 400 acres of chile peppers in 2015 for the US. The yield per acre was much higher for bell peppers at 376 cwt/acre compared with chile peppers at 223 cwt/acre. Overall US crop value in 2015 for bell peppers was $806M while the value for chile peppers was $135M.^1^ The past 20 years have shown a steady trend in the increase in production of peppers worldwide (2.9% per year in area harvested) as reported by the Food and Agriculture Organization of United Nations statistics service (2013, production data available at http://faostat3.fao.org/faostat-gateway/). Peppers are grown in at least 44 countries all over the world and as the worldwide population increases, demand for food and vegetable crops grows, whereas available land and other resources decrease. These conditions require a revolution in breeding technologies for pepper and vegetables in order to deal with these daunting challenges.

Most pepper species are diploid and have 12 pairs of chromosomes (2*n*=2×=24) which makes the *Capsicum* species amenable to traditional breeding methods as they are inter-fertile to varying degrees owing to their similar genome structures. The pepper genome is quite complex and contains a large amount of repetitive DNA sequences, which has caused inflation in the genome size to around 3.25–3.48 Gb^2,3^ compared with many other solanaceous species with genomes from 0.85–1.2 Gb.^4^ Because of the large genome size of pepper, development of sequence-based resources have been delayed compared with other Solanaceae, which have significantly smaller genome sizes, such as tomato (900 Mb) and potato (844 Mb), which had genome sequences released in 2012 and 2011, respectively.^5,6^

At the advent, the price for sequencing was very expensive, so discovery and analysis of the large pepper genome started with investigation of the gene space. Using 454 sequencing technology the first transcriptomes were developed in pepper using RNA from the fruit of two pepper parental lines CM334, Taean and their resulting hybrid.^7,8^ These were quickly followed by another assembly of unigenes that were derived from Sanger sequences of a hot pepper, Bugang.^9,10^ With a full transition to next-generation sequencing and the resulting marked reduction in sequencing cost and increase in output, discovery and analysis of the pepper genome moved from gene space-based studies to large scale genomic analyses. The Ashrafi *et al.*^9^ publication led this transition with the inclusion of three next-generation-sequencing-based assemblies. Genomics then jump started a new era in pepper breeding with the recent release of multiple *C. annuum* draft genome sequences including the Zunla-1 and varieties of *C. annuum* var. galbriusculum,^11^ the hot pepper landrace CM334, and *C. chinense*.^2^ These efforts also produced more transcriptome sequences in order to assist in annotation of the genomic sequences.

The transcriptome and genome assemblies make it possible to assess genetic differences and the structure of pepper germplasm for subsequent utilization in improvement of the crop. It is desirable to have methods to systematically assess diversity within *Capsicum* in an efficient and reproducible manner that will provide a large number of markers. Single-nucleotide polymorphism (SNP) markers are the preferred marker type as they have the possibility of occurring randomly throughout the genome, have a known sequence, and have the possibility to be the causative allele for beneficial phenotypic traits. With advancements in microarray technology, it is possible to perform high-density genotyping of SNPs in an extremely high-throughput manner, based on fixed probes on the microarrays. The unigene assembly produced by Ashrafi *et al.*^9^ was used to generate the first high-density system for analyzing SNPs in Capsisum in the form of an Affymetrix GeneChip.^10^ Hill *et al.*^10^ were able to assess the diversity among 40 *C. annuum* lines representing the primary breeding germplasm plus three additional species. However, this tool did not allow for identification of the specific SNP genotype at polymorphic loci and is no longer available publicly. A current publicly available tool for assaying a large number of loci with the ability to obtain exact genotypes would be a benefit to the pepper breeding community. Owing to the large genome size of pepper, whole-genome sequencing strategies that avoid large amounts of missing data would be costly and are usually unnecessary for QTL studies.^12^ In order to assist the pepper breeding community, we describe here the development of the PepperSNP16K array with one marker per annotated gene, and validation of the array by genetic mapping and diversity analysis for large-scale genotyping in pepper.

## Materials and methods

### Resequencing and SNP development

Leaf tissues from 22 lines representing multiple different chile and bell phenotypes of *C. annuum* were obtained and DNA was collected using the Qiagen (Valencia, CA, USA) DNeasy plant kit following manufacturer’s protocols, including RNase digestion. All DNA was quantified using Picogreen (Thermo Fisher Scientific, Waltham, MA, USA) and then prepared for sequencing using the Illumina TruSeq library preparation kit (FC-930-1023). Library products were size-selected for insert sizes ranging from 300–500 bp with Agencourt AMPure XP beads (Beckman Coulter, Indianapolis, IN, USA). The final libraries were quantified and analyzed on the Agilent Bioanalyzer (Santa Clara, CA, USA). Libraries were sequenced on the Illumina HiSeq2000 to generate 2×100 bp paired-end reads.

Adapters as well as 13 bases from the start and the last 5 bases of each read were removed from all raw reads due to poor quality. The remaining read sequences were quality trimmed and any reads fewer than 40 bases were removed using CLC Genomics Workbench 7.0 (https://www.qiagenbioinformatics.com/). Reads were aligned to the Pepper genome v1.5^2^ using CLC with default parameters. The CLC mapping files were exported as BAM files and were used in SAMtools^13^ as follows. In order to generate a file with all SNPs between all samples, SAMtools was used to merge BAM files for all samples to make a master BAM file. The merged file was sorted and then analyzed with SAMtools pileup (-cv) with SAMtools version 0.1.7a to call all variants in the merged file with the entire set of samples. Then variants are called for each individual sorted BAM file also with SAMtools pileup and options -c and -l. These resulting 22 files were then parsed and processed with a pipeline of in-house Perl scripts (Supplementary File 1) in order from 01 to 05 and run sequentially. To compensate for errors in sequencing and base calls, if a base represented >90% of reads, it was considered homozygous. This correction was required due to an issue that we identified with the SAMtools version that we used. In the pileup file of SAMtools (version 0.1.7a) for diploid genomes, a variant position is a non-reference allele from all the reads that are interrogating that putative variant position. We empirically found that at 90%, the call for variant is correct in 78–90% of the times depending on the crop (highest in pepper).^9^ After all individual pileup files were parsed and corrected, they were merged one-by-one to the parsed master pileup file to make a final Genotypes Table file that contained all polymorphic positions within the samples. Subsequently, the SNP caller script was run with Genotype Table file as input, requiring at least a set of two genotypes to be homozygous and different from each other with a minimum depth of three reads for each. After identifying all SNPs that met these criteria, the overall data set was filtered to remove SNPs that had nearby adjacent SNPs in the vicinity of 50 bases.

The filtered file with a list of positions for SNPs was used to categorize SNPs into three different sets, representing SNPs that (1) were found within blocky type peppers, (2) were found within hot type peppers and (3) were found to occur within both blocky and hot type peppers. The annotation information for Pepper version 1.5 was used to determine which SNPs were located in genes throughout the reference and used to annotate the determined SNPs.

### Array design

The upstream 50 bases and downstream 50 bases from each SNP in the set were obtained. These sequences were submitted to the Illumina Assay Design Tool (ADT) to obtain a design score for the Illumina Infinium technology. Infinium utilizes a bead-based array for genotyping with single base pair extension from a 50 bp oligonucleotide probe to assay the SNP base with fluorescently labeled nucleotides. The technology utilizes two fluorophores, therefore there are marker types that can be assayed with one-bead type (Infinium II) and other markers that require two-bead types (Infinium I). The markers were filtered to remove those with design score <0.8 and to select a single one-bead type marker (Infinium II) per gene across the Pepper v1.5 genome^2^ in order to obtain 19 000 markers with >9% minor allele frequency (2 out of 22) in the sequenced samples. A total of 2909 markers were selected from the blocky type data set; 3622 markers were selected from the hot type data set; and 12 469 markers were selected from the set within both blocky and hot types. Of the 19 000 SNPs targeted for manufacture of the array, 16 405 were represented following manufacture quality control resulting in the PepperSNP16K array.

### Genotyping with the array

Genomic DNA was fluorescently quantified with Picogreen or other assays to standardize samples at 50 ng μl^−1^ for each of the 84 lines representing a diverse germplasm background. Of the lines, 78 individuals were hybrid varieties and 6 individuals were inbred lines, which included the parents of known mapping populations. The hybrids were provided by a number of breeding companies in coded form but for most of the material, a description of the type of the respective line/variety was provided. For mapping, the parents of a cross between a hot type (Tabasco) and a blocky type (P4) described in Nagy *et al.*,^14^ and 90 individuals of an F_2_ mapping population were used. Samples were processed according to Illumina instructions and hybridized to the PepperSNP16K array and the arrays were analyzed with the Illumina iScan to measure fluorescent genotyping data. All image files were uploaded into a single GenomeStudio project containing the 177 individuals. Data from all markers were clustered using GenomeStudio Genotyping Module (v1.9.4, Illumina, Inc., San Diego, CA, USA). All markers were then viewed and manually curated, for construction of an optimized cluster file for pepper. The cluster file is available at http://www.solgenomics.net. Genotypes for each sample were exported using the final cluster file and utilized in further analyses. The quality of markers and their minor allele frequencies were determined.

### Genetic linkage analysis

Genotypes from both parents (Tabasco a hot type and P4 a blocky type), F_1_ and 90 individuals were obtained. The data were transformed into mapping data format (‘ABH’) for markers that were found to be segregating in a co-dominant pattern. The mapping data format files were used to map in JoinMap v4.0^15^ using default parameters with respect to grouping and ordering. The map orders were exported and haplotype maps were examined to manually remove problematic markers which were elevating double crossover numbers and increasing map size. After removing the problematic markers, the final linkage groups were generated in Map Manager QTX^16^ with no prior assumptions on marker position using the following settings: linkage evaluation F_2_, search linkage criterion *P*=0.05, map function Kosambi, and cross-type line cross. The resulting order of markers were validated using CheckMatrix software (www.atgc.org/XLinkage/). Any linkage groups that produced errantly ordered markers were manually reanalyzed for problematic markers, which were subsequently removed before repeating the process to determine the final orders. The final maps were drawn with MapChart version 2.2.^17^

The positions in the linkage map compared with the marker position on the Pepper reference genome (v1.5) were plotted. The positions of all markers on the array on the updated Pepper reference genome V1.55 (http://peppergenome.snu.ac.kr/) were determined using the SNP and flanking sequence by BLAST alignment. The correlation of position on the linkage map to the updated reference version 1.55 was also plotted. Correlation with the interspecific map of a cross between *C. frutescens* accession BG2814-6 x *C. annuum* ‘NuMex RNaky’ (FA map) produced by Hill *et al.*^18^ was analyzed based on markers positioned in shared genes across the two technologies. Linkage map positions in each of the respective maps were plotted to determine the *R*^2^ between the two maps. Correlation with the (FA) map was used to orient the linkage groups. Array markers and linkage map positions are available on the Sol Genomics Network database (https://solgenomics.net).

### Analysis of population structure

We used the software package STRUCTURE to reveal the population structure of the investigated pepper lines and hybrids.^19^ For the SNP marker set, STRUCTURE was run for *K*=1–20. For each value of *K*, five replications were performed with a length of burn-in period of 100 and 1000 MCMC reps after burn-in. To determine the most probable value of *K*, we applied the *ad hoc* criterion described by Evanno *et al*.^20^

## Results

### Array development

Coverage for each sample ranged from 19- to 80-fold for each of the resequenced pepper lines. Once these reads were processed, an average depth of 12.9× was mapped back to the Pepper reference genome v1.5 covering an average of 86.8% of the genome. The mapped reads were utilized with SAMtools and an in-house pipeline to identify SNPs. The Illumina Infinium array was designed with 19 000 putative SNP markers, of which 16 405 SNPs (86.3%) passed through the manufacturing process with adequate quality and representation to be included in the manifest. This set is composed of 2531 SNPs, which are unique within blocky types, 3058 SNPs, which are unique within hot types, and 10 816 SNPs, which are found within both hot and blocky types. The position of SNPs along Pepper genome version 1.5 were utilized to annotate those SNPs that fell within predicted genes (Supplementary Table 1). The proportion of individuals that had adequate signal for a marker making it amenable to genotyping, or ‘call frequency’, was calculated (Table 1). A total of 1528 or 9.3% of markers were deemed as functionally failed due to the inability to call any samples for these markers. This resulted in the remaining 14 877 markers being classified as ‘functional’. These functional markers were then classified into two groups, those which represented monomorphic loci in which no difference in genotype was seen among samples assayed, and those which showed differences and were thus polymorphic. A total of 13 800 markers of the 16 405 assays on the array were found to be polymorphic, which is a success rate of 84.1% for the array overall with our samples. The 1077 functional but monomorphic markers could either be falsely identified SNPs or are polymorphisms not represented in our germplasm run on the array. The GenTrain score of the polymorphic markers was assayed to determine the behavior of the clusters in the genotype plots, with 1 representing very well-discriminated and tight clusters. The GenTrain scores for all polymorphic markers are shown in Table 1 and, as expected for the diploid pepper genome, the majority of the markers exhibited very high GenTrain scores. The minor allele frequency observed within the sample set was also calculated for all polymorphic markers (Table 1). This showed that there were 13 760, 12 655, 11 965 and 9921 SNPs represented for minor allele frequencies of greater than 1%, 3%, 5% and 10%, respectively. The 14 877 functional markers comprise the PepperSNP16K array. The resulting cluster file for the array is available at http://www.solgenomics.net.

### Genetic map construction

**Figure 1:**
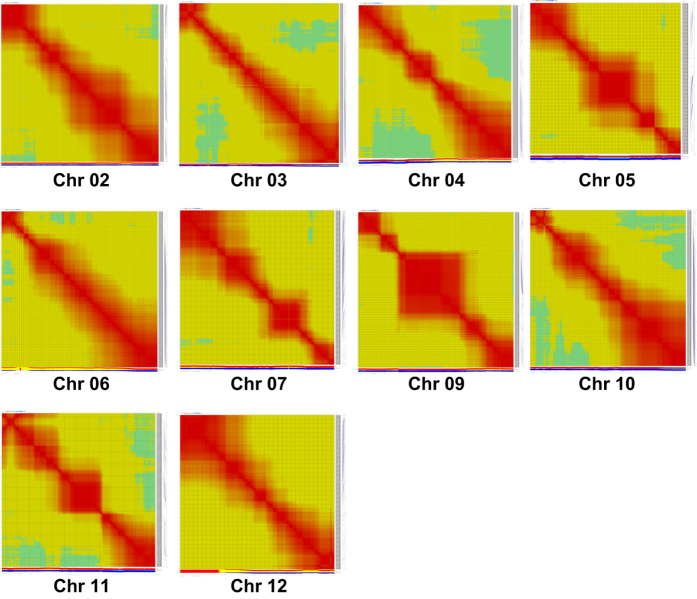
Heat map plots produced with CheckMatrix software for genetic map orders of all chromosomes, except Chr01/08. All orders produced from initial mappings except for Chromosome 07, which is shown after manual removal of problematic markers.

**Figure 2:**
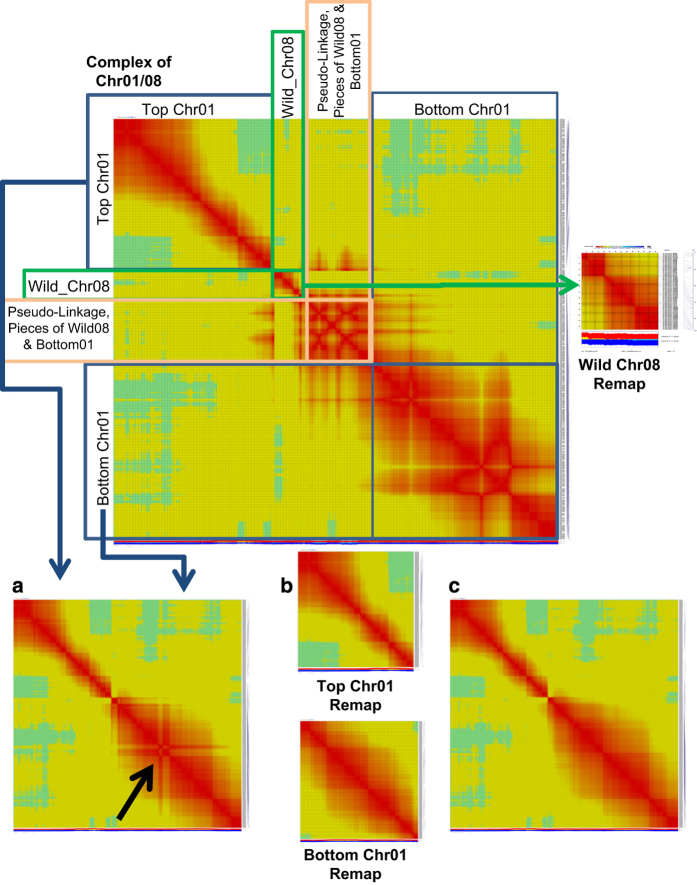
Initial ordering and subsequent ordering of the separate pieces of the Chromosome 01 and 08 complex due to translocation between *C. annuum* and *C. frutescens* parents. Remapping of Chromosome 01 by (**a**) reordering all markers from both top and bottom pieces together and (**b**) mapping top and bottom separately and (**c**) calculating distances between markers with a set order based on the mapping of the top and bottom pieces separately.

**Figure 3:**
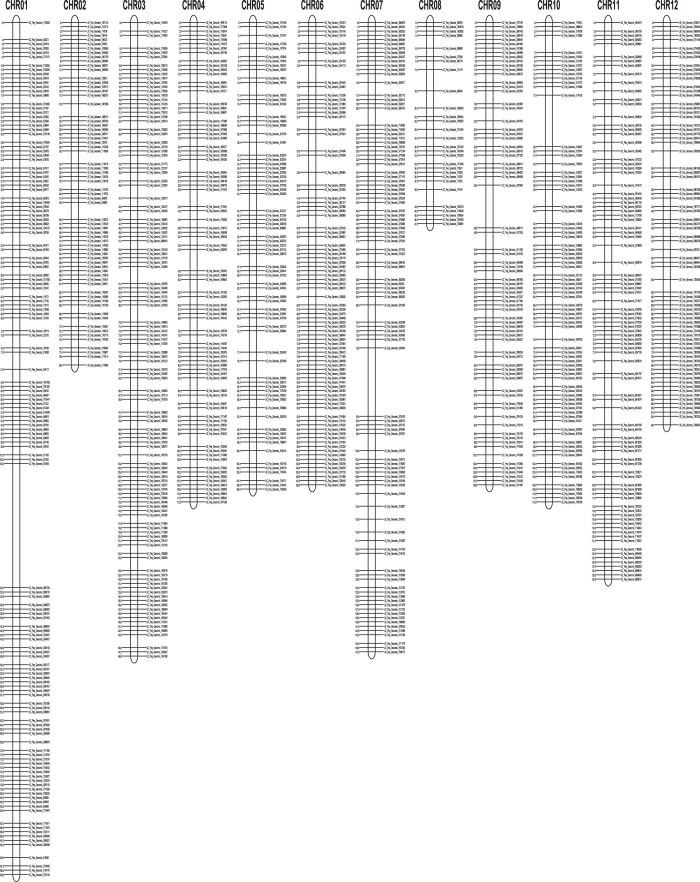
Interspecific linkage map of the 12 pepper chromosomes. Map determined using 90 F_2_ individuals from a cross between a Tabasco (hot type parent), and P4, a blocky type parent. Only one marker is listed on the right per centiMorgan, even if there were more markers co-segregating.

The initial genetic map generated with the array was created with an F_2_ population derived from a cross between a hot pepper and a sweet pepper parent (Tabasco x P4). Most linkage groups easily fell into place and generated expected heat map plots across the resulting order; however, three chromosomes (Chr01, Chr07 and Chr08) needed manual adjustment following visualization of the initial orders determined using CheckMaxtrix (www.atgc.org/XLinkage/). After removal of a few problematic markers, the software was accurately able to determine the ordering for Chr07 (Figure 1). However, Chr01 and Chr08 were problematic due to a translocation event that is present between the parents in this interspecific cross. As this is the case, each of the pieces as depicted by Hill *et al.*^18^ were broken out according to their groupings based on LOD score, and the groups were identified based on overlap with the FA map in that study and overlap with the draft genome assembly. The groups identified as the top of Chr01, the bottom of Chr01, wild Chr08 and a combination of both Chr01 pieces were ordered independently and visualized with CheckMatrix (Figure 2). It was observed that when the top and bottom pieces of Chr01 were attempted to be ordered together, the software generated a non-optimal ordering of the markers (Figure 2a). Owing to this, the order from the independent ordering of each piece was used to establish a final order (Figure 2b), then distances between each marker were calculated for that set order (Figure 2c). Finally, a total of 5546 markers were mapped into 12 linkage groups representing a total of 1392.3 cM (Figure 3). These represent largely markers from the set found within both blocky and hot types (3963). However, as the parents were not included in the set of samples sequenced for marker development and classification of markers into phenotype sets, the population was also able to map a number of markers from the unique sets including 713 from the blocky set and 918 markers that were from the hot set. As the marker classification is dependent on the samples used, in this case, based on the lines sequenced, the classifications that are provided in the ‘Type’ column in Supplementary Table 1 should be utilized loosely.

**Figure 4:**
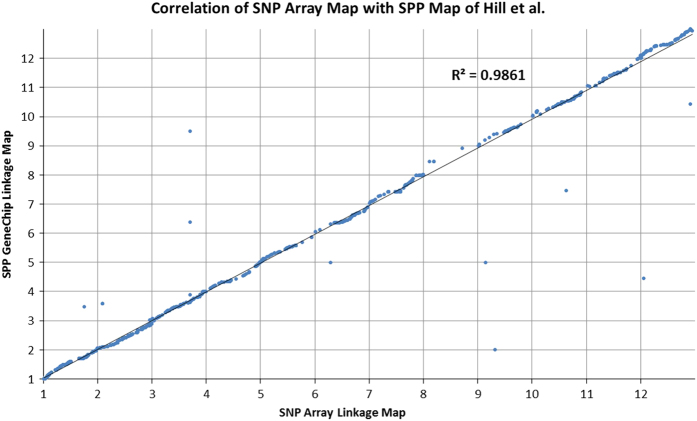
Correlation of interspecific map with FA map produced by Hill *et al*.^18^ Positions across linkage groups are normalized. Numbers along each axis corresponds to each pepper chromosome.

**Figure 5:**
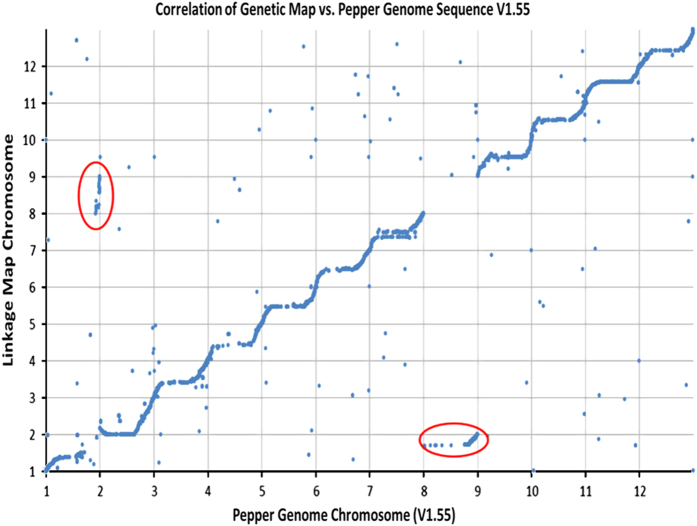
Correlation of interspecific linkage map with pepper draft genome sequence version 1.55.

This map corresponds to 1361 unique recombination bins with an average of 113 bins per chromosome. On average there are 462 markers on each linkage group. This translates to a marker on average across the genome every 610 Mb or 0.25 cM, based on chromosome sizes in Pepper Genome Version 1.5. Due to the translocation between the parents, Chr01 represents the largest map distance at 199.1 cM, whereas Chr08 represents the smallest map distance at 46.7 cM, which is consistent with the findings of Hill *et al*.^18^ Overall the FA map produced by Hill *et al.*^18^ and the map produced here are exceptionally similar with an* R*^2^ of 0.9861 (Figure 4) based on 822 markers in shared genes. Most chromosomes show a high number of overlapping markers, except for the smallest, chromosome 08, that is also genetically the shortest, contains the least number of markers and can be seen in Figure 5 to have only a few overlapping markers. The total sizes were also highly similar at 1380 cM for the FA map and 1392 cM in the map produced with the PepperSNP16K array.

### Synteny to reference genome sequences

Most linkage groups from the interspecific genetic map showed a very good correlation with the most recent version (1.55) of the pepper draft reference genome (Figure 5) as well as the original published version (Supplementary Figure 1); however, not all loci were able to be translated to version 1.55 from version 1.5 (Supplementary Table 1). Generally, the order of markers between original version 1.5 and the updated version 1.55 was highly similar with *R*^2^ of 0.9754 (Supplementary Figure 1). It was also observed that chromosomes 01 and 08 that were involved in a translocation between the two parents of the population, show association with the opposite chromosome from the draft reference sequence, which are circled in the figures. Chromosome 07 which initially caused some problems in ordering, again shows abnormalities when mapped back to the reference recurrence. It is possible that there are some changes in genome structure between the parents in this chromosome as well that may be causing these issues with ordering and what is observed back to the CM334-based reference, as CM334 is a landrace in *C. annuum*.

### Diversity analysis

**Figure 6:**
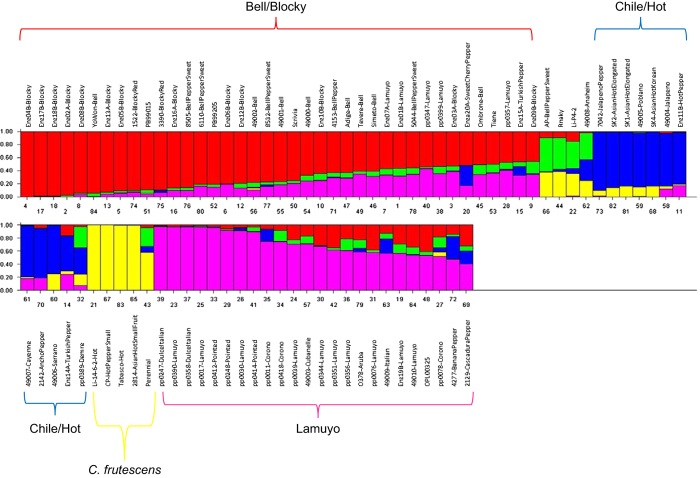
STRUCTURE analysis of inbred pepper lines using the data produced with the PepperSNP16K array analyzed in STRUCTURE for *K*=5.

As expected, the distinction between the two pepper types can be clearly visualized by STRUCTURE analysis (Figure 6) utilizing 11 027 polymorphic SNPs for the analysis after filtering. The analysis with respect to the *K *value revealed a maximum value between 4 and 5. Thus, we have used a *K* value of 5 to analyze the population structure and evaluate the results with respect to known information about the type and species classification of the material.

The subgroup splits are generally observable along distinct fruit types. Assessing the five groups based on the available knowledge concerning the material, the red and pink groups are blocky types while the green and blue groups represent the chile/hot types. The largest group A (red) is represented by mainly bell or blocky types together with a limited set of Lamuyo types. The second largest group B (pink) is mainly represented by Lamuyo, Pointed, Corono and Italian types. Although there is some overlap with group A, this group B is sufficiently distinct from the A group. The third largest group C (blue) is represented by mainly hot types including a considerable number of Asian hot types. The next group D (yellow) is represented by hot pepper such as Tabasco that are different species (*C. frutescens*) or appear to contain a considerable proportion of exotic introgressions. The fifth and smallest group E (green) contains varieties that are *C. annuum* probably with significant introgressions from wild species. It is known that in pepper, specific disease resistance genes have been introgressed from other species. In summary, the classification described in the STRUCTURE analysis is mainly in agreement with what is known about the different pepper types and the data from microsatellite analysis,^14^ where similar groups could be identified and confirmed through lines that were analyzed in both studies. The results also correlate with the analysis completed with a smaller set of 40 lines analyzed with the Affymetrix GeneChip.^10^

**Figure 7:**
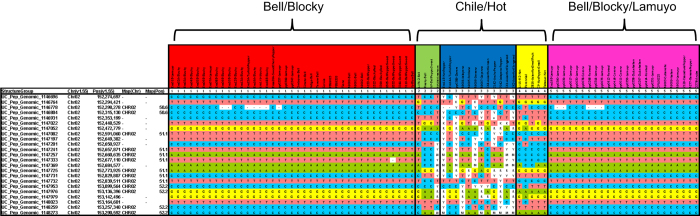
Monomorphic haplotype block in non-pungent inbred types surrounding PUN1 locus. Germplasm groups depicted were determined with the STRUCTURE program.

The genotypes of lines were used to identify the non-polymorphic region surrounding the *PUN1*/*CSY1* gene (Ca02g19260) by correlating the STRUCTURE analysis according to phenotypic group with the SNP markers around the gene. Although a marker specifically in the pun1 gene has not been included on the Pepper16K array, the physical location surrounding the genome position of the identified pun1 locus in the draft genome sequence revealed 23 SNPs for which the non-pungent group types identified using STRUCTURE are monomorphic. This region was found to correspond to a physical distance according to the genome sequence of 1.016 Megabase pairs and a genetic distance of 1.6 cM, spanning from 50.6 to 52.2 cM on Chromosome 02 (Figure 7).

## Discussion

We have developed a publically available high-throughput, high-density SNP genotyping platform for pepper, the PepperSNP16K array. Interrogation of the 14 877 functional markers on the array allowed the production of the first interspecific genetic map and the first small-scale germplasm analysis using the array. This resource will allow for researchers as well as breeders to obtain easy-to-work with, reproducible data with very low rates of missing data that can be directly integrated into their breeding platforms through marker-assisted selection and discovery of markers associated with economically important traits for pepper.

The array will be valuable for investigating the *Capsicum* genus with limited ascertainment bias, but it must be taken into consideration that while many species have the same chromosome number and are interfertile among the genera, significant differences in genome/chromosome structure can be present that will create difficulties when attempting to create a genetic map with a population generated with interspecific parents. This was seen in the interspecific cross used here between *C. frutescens* and *C. annuum* that created varying amounts of difficulties for three chromosomes. As previous researchers have demonstrated, despite the occurrence of a translocation in *C. frutescens* relative to *C. annuum*, it was possible to identify the groupings of markers that corresponded to the two separate pieces of chromosome 01, which correspond to the top of chromosome 01 and the bottom translocated region, which corresponds to cultivated chromosome 08 and the wild chromosome 08 that corresponds to the bottom of cultivated chromosome 01 (as outlined in Hill *et al.*^18,21^—Figure 6). Hill *et al.* reported that there is a pseudolinkage region that occurs between chromosomes 01 and 08 in the interspecific cross, which caused issues with local ordering of markers within that region. In their case, they had about three times the amount of markers included in their genetic map, which allowed for them to generate a consecutive linkage block that corresponded to chromosome 01 and showed pseudolinkage to chromosome 08. Although with a smaller number of markers, we were unable to generate a consecutive linkage group for chromosome 01, so it was necessary to break it into two pieces that were ordered independently, then joined to determine the final distances between the markers over the entire chromosome (Figure 2).

The results and correlation with the Hill *et al.*^18^ map were extremely valuable in this study in order to assist with issues with problematic chromosomes involving the translocation; however, there are a number of differences between the arrays used in the two studies. In the previous study a GeneChip array was utilized in order to identify single-position polymorphisms (SPPs); although this approach offers the possibility to discover *de novo* markers in the same way as through sequencing, there are limitations to SPPs relative to SNPs. Calling of SPPs is susceptible to both missing and erroneous calls owing to the algorithm/technology not being able to discriminate heterozygous calls,^10^ although the degree of redundancy (13 probes per bp) resulted in highly accurate calls in RIL populations. Therefore, for individuals or mapping populations such as F_2_s that have a significant proportion of heterozygous loci, these positions will have to be corrected prior to direct utilization of the data. The correction of these issues can lead to downstream problems as association software or genetic mapping/ordering of markers is highly susceptible to calling errors, particularly in high-density maps. In addition, a discrete genotype is not obtained by calling of SPPs, just the relative hybridization intensity is obtained, not the actual genotype at the locus that can be obtained with SNP genotyping methods. Determination of ‘genotypes’ from the GeneChip array that delivers just hybridization intensities requires a downstream analysis following actual reading to infer the genotype, whereas in the Infinium analysis, the genotype is obtained directly following reading of the SNP array. These differences make processing of the GeneChip array computationally intensive and time consuming following obtaining results from the laboratory, while the output from the SNP arrays are ready for analysis directly following data acquisition.

Post-processing of genotyping-by-sequencing and other genotyping through sequencing methods also require bioinformaticians with knowledge of the data to process from raw data to genotypes and can take weeks to months. Once genotype data are obtained, the missing data rates are quite high, utilized data for published analyses are typically up to 17–20%.^22–24^ In order to achieve acceptable missing rates for downstream analyses, imputation algorithms have to be utilized. However, the accuracy of imputation methods can be detrimental to downstream analyses, which depend on good genotypic data.^25^ For this reason the low missing rates produced with array technology make utilization of the genotyping data produced straightforward. The genotyping rate achieved with the PepperSNP16K array was 99.2% for functional polymorphic markers, thus producing a missing rate of <1% overall. Similar genotyping rates have been seen with other recently produced arrays for cotton,^26^ soybean^27^ and rice.^28^ Similar challenges with calling heterozygotes are found in GBS data due to low coverage in sequencing each base.^25^ The SNP arrays thus allow for high-quality, high-density genotype data that will be ready for analysis from plant material within 72 h.

The quick turnaround from plant material to high-quality data can be a valuable tool for breeders when the applicability of the data to breeding programs is understood. *De novo* clustering of markers into the haploid number of chromosomes in pepper maps has previously been rare.^18^ The array has been shown to accurately separate polymorphic markers into 12 linkage groups corresponding to the haploid number of chromosomes in pepper. The approach to use a selection of gene-associated SNPs on the array should provide a fairly even distribution of markers along the genome as genes have been shown to have a distribution that is similar to the distribution of genetic recombination and across the reference sequence.^2^ Analysis of the 84 hybrid and inbred lines genotyped on the PepperSNP16K showed that the available germplasm in *Capsicum* is largely divided based on phenotypic characteristics of the fruit. In this study, the germplasm was shown to split into five distinct groups. The available diversity within each phenotypic group appears to be fairly variable, which will allow for improvement of groups through interbreeding within a phenotypic class.

Although diversity within groups is still available, diversity within specific regions responsible for particular traits may be fixed within the group, which can create difficulties for breeding for traits controlled by loci in such areas within specific populations. One such genomic area that accounts for one of the most important traits for breeding in the *Capsicum* genus is at the *PUN1*/*CSY1* gene (Ca02g19260). This locus is responsible for the presence of capsaicin in pungent varieties (chile/hot) or lack of capsaicin in non-pungent (bell) varieties.^29,30^ The gene is located on Chromosome 02 at 152.61 Mb on version 1.55 of the pepper draft genome sequence.^2^ Surrounding this locus Hill *et al.*^10^ was able to identify 42 markers that were monomorphic among non-pungent lines, which extended to over 8.74 cM on Chromosome 02. Here we were able to reduce the region down to a ~1 Mb region corresponding to just over 1.6 cM of genetic distance. It is likely that the inclusion of additional samples in the diversity analysis shown here compared with the previous investigation was able to reduce the size of the region likely affected by the selective sweep surrounding the *PUN1* gene and the phenotypic split between pungent and non-pungent pepper types. In breeding populations, it will be important to utilize population scale data to identify regions with low diversity, if there are important genes which may attribute beneficial characteristics in those areas. If so, genetic diversity will need to be taken in from outside sources to allow for inclusion of diversity within these areas.

Datasets such as the one that was developed here will allow for breeders to have additional population-scale information for addressing breeding at the genetic scale for such key low diversity areas. The genetic map produced will also allow for localization of 4724 additional genes on the genetic map that were unable to be localized in previous high-density mapping efforts, which will allow for progression of fine mapping efforts for genes responsible for agronomically important traits.

## Conclusions

We describe a publicly available standardized high-throughput genotyping array for pepper, the PepperSNP16K. This array and the accompanying resources developed here will be a significant addition to the pepper breeder’s toolbox which will allow them to focus on improving pepper in a time of growing demand and decreasing resources. Due to the standardized nature of the array, research using the array will occur on a worldwide common platform that will permit assimilation of results on a common reference tool. This will allow the deployment of findings for marker-assisted breeding programs worldwide.

## Figures and Tables

**Table 1:** Validation statistics for markers on the PepperSNP16K array based on genotyping of 84 inbred and hybrid lines for (a) call frequency for all markers on the array, (b) marker clustering statistics or GenTrain score for all polymorphic markers and (c) minor allele frequencies for all polymorphic SNPs determined using 84 inbred and hybrid lines; mapping samples were not included.

	*Marker*	
	*Count*	*Percentage*
(a) Call frequency		
0.000–0.750	1579	9.63
0.750–0.980	1688	10.29
0.980–0.999	2742	16.71
1.000	10 396	63.37
Total	16 405	100.00
		
(b) GenTrain Score		
<0.2	71	0.51
<0.3	684	4.96
<0.6	2426	17.58
<0.8	4139	29.99
>0.8	6480	46.96
Total	13 800	100.00
		
(c) Minor allele frequency		
0.00–0.05	1835	13.30
0.05–0.10	2044	14.81
0.10–0.15	1990	14.42
0.15–0.20	1491	10.80
0.20–0.25	1285	9.31
0.25–0.30	1192	8.64
0.30–0.35	989	7.17
0.35–0.40	1051	7.62
0.40–0.45	985	7.14
0.45–0.50	938	6.80
Total	13 800	100.00
