# Supplementary figures and images for: A HapMap leads to a Capsicum annuum SNP infinium array: a new tool for pepper breeding
Source: Hortic Res. 2016 Jul 27;3:16036–. doi: 10.1038/hortres.2016.36 (PMC4962762; doi:10.1038/hortres.2016.36)

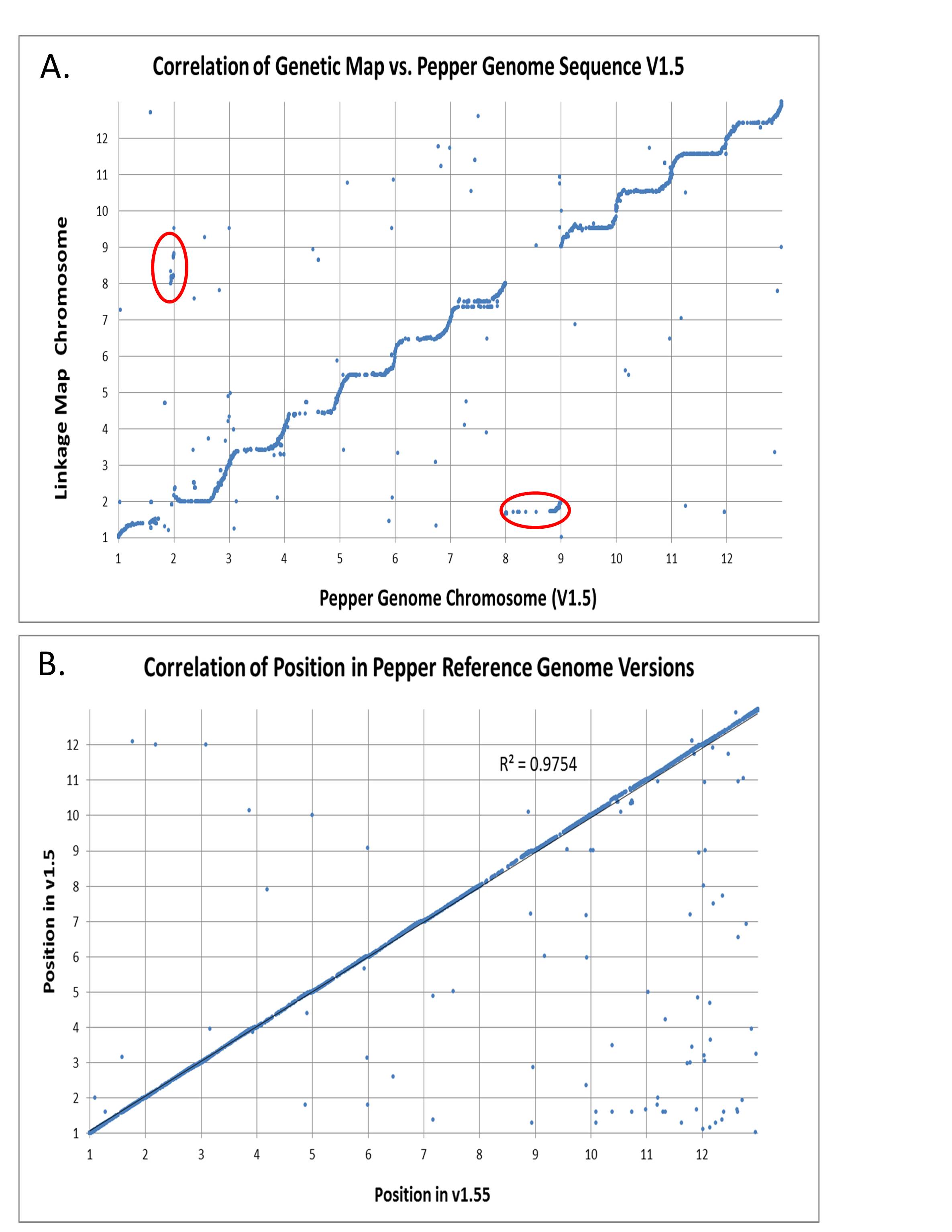

Supplement: Supplementary Figure 1 [file hortres201636-s1.jpg]
